# Fusogenic vesicular stomatitis virus combined with natural killer T cell immunotherapy controls metastatic breast cancer

**DOI:** 10.1186/s13058-024-01818-5

**Published:** 2024-05-15

**Authors:** Adam Nelson, Nichole McMullen, Simon Gebremeskel, Roberto De Antueno, Duncan Mackenzie, Roy Duncan, Brent Johnston

**Affiliations:** 1 Department of Microbiology and Immunology, Dalhousie University, B3H 4R2 Halifax, NS Canada; 2 Beatrice Hunter Cancer Research Institute, B3H 4R2 Halifax, NS Canada; 3 Department of Biochemistry and Molecular Biology, Dalhousie University, B3H 4R2 Halifax, NS Canada; 4 Department of Pediatrics, Dalhousie University, B3H 4R2 Halifax, NS Canada; 5 Department of Pathology, Dalhousie University, B3H 4R2 Halifax, NS Canada

**Keywords:** Natural killer T cells, Vesicular stomatitis virus, Triple negative breast cancer, Fusion associated small transmembrane proteins, Adoptive transfers, α-galactosylceramide, Anti-tumor immunity, Oncolytic viruses, Immunotherapy

## Abstract

**Background:**

Metastatic breast cancer is a leading cause of cancer death in woman. Current treatment options are often associated with adverse side effects and poor outcomes, demonstrating the need for effective new treatments. Immunotherapies can provide durable outcomes in many cancers; however, limited success has been achieved in metastatic triple negative breast cancer. We tested whether combining different immunotherapies can target metastatic triple negative breast cancer in pre-clinical models.

**Methods:**

Using primary and metastatic 4T1 triple negative mammary carcinoma models, we examined the therapeutic effects of oncolytic vesicular stomatitis virus (VSVΔM51) engineered to express reovirus-derived fusion associated small transmembrane proteins p14 (VSV-p14) or p15 (VSV-p15). These viruses were delivered alone or in combination with natural killer T (NKT) cell activation therapy mediated by adoptive transfer of α-galactosylceramide-loaded dendritic cells.

**Results:**

Treatment of primary 4T1 tumors with VSV-p14 or VSV-p15 alone increased immunogenic tumor cell death, attenuated tumor growth, and enhanced immune cell infiltration and activation compared to control oncolytic virus (VSV-GFP) treatments and untreated mice. When combined with NKT cell activation therapy, oncolytic VSV-p14 and VSV-p15 reduced metastatic lung burden to undetectable levels in all mice and generated immune memory as evidenced by enhanced in vitro recall responses (tumor killing and cytokine production) and impaired tumor growth upon rechallenge.

**Conclusion:**

Combining NKT cell immunotherapy with enhanced oncolytic virotherapy increased anti-tumor immune targeting of lung metastasis and presents a promising treatment strategy for metastatic breast cancer.

**Supplementary Information:**

The online version contains supplementary material available at 10.1186/s13058-024-01818-5.

## Introduction

Breast cancer is the most common cancer, and the second leading cause of cancer death in women [1]. Triple negative breast cancer (Her2^neg^, estrogen receptor^neg^_,_ progesterone receptor^neg^) has the lowest survival rate of any breast cancer subtype due to a lack of targeted therapies and high rates of metastasis [2, 3]. Consequently, metastasis is the leading cause of breast cancer related deaths [4]. Current therapeutic treatments, including chemotherapy and radiation, often mediate suboptimal responses and can cause serious adverse events [5, 6]. Furthermore, mutations to proteins involved in drug uptake and metabolism often lead to acquired resistance that reduces chemotherapeutic efficacy over time [7]. These considerations highlight the need for new therapeutic approaches to target metastatic triple negative breast cancer. This study examined an approach combining fusogenic oncolytic viruses with natural killer T (NKT) cell-based immunotherapy.

NKT cells are a conserved population of glycolipid-reactive T lymphocytes that play an important role in tumor immunosurveillance and control [8–10]. NKT cells express an invariant TCRα chain rearrangement (Vα14-Jα18 in mice and Vα24-Jα18 in humans) allowing them to recognize endogenous and exogenous glycolipids presented by CD1d [11]. NKT cells can be activated by free administration of the glycolipid antigen α-galactosylceramide (α-GalCer), but transfer of glycolipid-loaded dendritic cells (DCs) mediates superior anti-tumor responses [12–14]. Activated NKT cells exhibit cytotoxic function [13, 15] and rapidly generate cytokines that regulate the function of other immune cells [13, 16, 17]. Therapeutic administration of α-GalCer slows tumor progression in human patients [18] and NKT cell infiltration is associated with a good prognosis in several cancers including neuroblastomas [19], pancreatic [20], and colon cancers [21]. Previously, we demonstrated that NKT cell immunotherapy could effectively target breast cancer metastasis, resulting in complete clearance of metastases in 40–50% of mice [13]. Combining NKT cell immunotherapy with chemotherapy [22] or oncolytic vesicular stomatitis virus (VSV) [23] increased survival by an additional 20–25%. As chemotherapy is associated with dose-limiting toxicities and adverse effects [5, 6], we looked to improve the oncolytic virus component of our dual treatment regimen.

Oncolytic viruses preferentially infect and kill cancer cells via altered expression of viral entry receptors, defects in anti-viral defences, or alterations in cellular metabolism [24–26]. While oncolytic viruses can directly kill cancer cells, there is growing evidence that oncolytic viruses act to stimulate anti-tumor immunity [27–29]. Oncolytic VSV is an attenuated negative strand RNA virus from the *Rhabdoviridae* family engineered with a methionine deletion in its matrix protein (VSVΔM51). This inhibits the ability of VSV to block nuclear export of IFNβ mRNA and increases its sensitivity to type I IFNs [25, 30], thereby restricting replication to cancer cells, which commonly harbour defects in type I IFN signalling, and limiting off target effects [25]. Previous preclinical trials demonstrated that oncolytic VSV can improve cancer control when combined with different immunotherapies, including NKT cell immunotherapy [23, 29], checkpoint inhibitors [31], and chimeric antigen receptor T cell therapy [32].

The VSV genome is amenable to modification via reverse genetics, allowing for insertion of exogenous genes that increase the oncolytic or immunomodulatory capacity of the virus [33]. Previously, we demonstrated that VSV encoding the reovirus p14 fusion-associated small transmembrane (FAST) protein increased the efficacy of VSV monovirotherapy [34]. FAST proteins are the smallest viral membrane fusion proteins (∼ 100–150 amino acids) and are the only examples of nonenveloped virus membrane fusogens that induce syncytium formation [35], causing membrane fusion at neutral pH following their expression and trafficking to the cell membrane of infected cells [36]. Syncytium formation facilitates rapid, localized cell-cell transmission of the virus followed eventually by syncytial lysis [37]. FAST proteins do not bind to specific cell receptors and hence can fuse most cells, including cancer cells [38, 39]. We previously exploited the syncytium-inducing capability of the p14 FAST protein from reptilian reovirus to establish proof-of-concept that a syncytium-inducing VSV has enhanced oncolytic activity without increased toxicity [34].

In addition to reptilian reovirus p14, there are several other members of the FAST protein family that share limited sequence similarity and possess differing abilities to cause cell-cell fusion and syncytial lysis [37, 40]. Here, we compared the effects of p14 and two additional FAST proteins, the baboon reovirus p15 and avian reovirus p10 FAST proteins, on VSV syncytium formation and oncolytic virotherapy, alone and in combination with NKT cell immunotherapy in primary and metastatic breast cancer models. Results showed a correlation between the relative syncytiogenic activity in vitro and virotherapy efficacy of the different FAST proteins in vivo. The most highly syncytiogenic VSV-p15 construct was also the most effective at increasing immunogenic cell death, inhibiting tumor growth, prolonging survival, and enhancing tumor immune infiltration in an aggressive primary 4T1 breast cancer model. Most notably, lung metastases were undetectable in mice that received VSV-p15 or VSV-p14 combined with NKT cell immunotherapy, with VSV- p15 able to mediate this protection at a 10-fold lower viral dose. Thus, VSV expressing highly fusogenic FAST proteins enhance virotherapy and can be effectively combined with NKT cell immunotherapy to improve survival in a metastatic breast cancer mouse model.

## Materials and methods

### Mice

Female BALB/c mice were purchased form Charles River Laboratories. Mice were maintained in the Carleton Animal Care Facility at Dalhousie University and used at 8–12 weeks of age. Mice were group housed in temperature-controlled rooms with a 12-hour light/dark cycle.

### Cell culture

4T1 mammary carcinoma cells (CRL-2539), MCF7 human adenocarcinoma cells (CVCL_0031), and Vero kidney epithelial cells (CCL-81) were purchased from ATCC. Cell lines were cultured at 37 °C, 5% CO_2_ in DMEM supplemented with 10% FBS, 100 units/mL of penicillin (1%) (Hyclone), and 100 µg/mL of streptomycin (1%) (Hyclone).

### Breast cancer models

4T1 mammary adenocarcinoma cells were harvested in the logarithmic growth phase using trypsin-ethylenediaminetetraacetic acid (VWR). Cells were resuspended in PBS and 2 × 10^5^ cells (50 µL volume) were injected subcutaneously. For the primary subcutaneous model, 4T1 tumors were measured every second day using electronic calipers and tumor volume was calculated: volume = (W^2^ X L)/2. Primary tumors (∼ 200mm^3^) were injected intratumorally on days 10, 12, and 14 with either PBS, VSV-GFP, VSV-p10ARV, VSV-p14, or VSV-p15 (5 × 10^8^ or 5 × 10^7^ PFU/mouse). Tumor volumes were monitored over time. In some groups, spleens, blood, and tumors were isolated 24 h after the final virus injection for analysis of immune cells and immunogenic cell death markers.

For the metastasis model, tumors (∼ 200mm^3^) were surgically resected using aseptic technique on day 12. On days 13, 15, and 17 mice were injected intravenously with either PBS, VSV-GFP, VSV-p14, or VSV-p15 (5 × 10^8^ or 5 × 10^7^ PFU/mouse). On Day 18, mice were injected intravenously with PBS or bone marrow derived dendritic cells loaded with α-GalCer (2 × 10^5^) to activate and expand the NKT cells. Survival was monitored over time. Spleens and lungs were isolated 7 days post NKT cell activation for immune profiling assays.

### Generation of recombinant VSV

All virus constructs were generated using the attenuated VSVΔM51 platform, which has increased Interferon sensitivity and tumor tropism [25]. VSV-GFP and VSV-p14 were generated as previously described [34]. The p15 and p10ARV FAST genes were amplified by PCR and subcloned into the XhoI and NheI sites located between the G and L genes in pVSVΔM51-XN [25]. The recombinant VSV expressing p15 and p10ARV were rescued as previously described [34]. VSV encoding firefly luciferase (VSV-fluc) was provided by Dr. John Bell (University of Ottawa) [41].

### Virus purification

Vero cells at ∼ 95% confluency were infected with recombinant VSV viruses at a MOI of 0.1 in serum free DMEM for 48 h. Supernatants were collected and filtered through 45 μm filters. Each supernatant was layered on 1.1 ml of 20% sucrose in PBS and centrifuged at 160,000 x g for 90 min at 4 °C. Collected virus was resuspended in 15% glucose in PBS and stored in at -80 °C. Virus was titred on Vero cells by plaque assay.

### Syncytia imaging and nuclei quantification

4T1 cells were cultured with vehicle (uninfected) or infected at an MOI of 1 with VSV-GFP, VSV-p10ARV, VSV-p14, or VSV-p15. Syncytia were examined using a Nikon Diaphot Inverted microscope (magnification 10x, numerical aperture 0.25) 12.5 h after infection at room temperature, using water as an imaging medium. Images were acquired using a Nikon D300S camera using Camera Control Pro 2 software. To quantify nuclei per syncytia, 4T1 cells were infected at an MOI of 1 with VSV-GFP, VSV-p10ARV, VSV-p14, or VSV-p15. Cells were fixed after 15 h and stained with Giemsa. Syncytial nuclei and total nuclei were counted, and the percent fusion index (F_index_) was calculated: %F_index_ = (syncytia nuclei/total nuclei) X 100%.

### Bone marrow derived dendritic cells

To generate bone marrow derived dendritic cells, bone marrow was extracted from the femur and tibia of 8–12-week-old female BALB/c mice and cultured in 6-well plates in complete RPMI-1640 (10% FBS, 50 µM 2-mercaptoethanol, 2 mM L-glutamine, 1X non-essential amino acids, 1mM sodium pyruvate, 100 µg/mL streptomycin, and 100 units/mL penicillin) with 40ng/mL of GM-CSF and 10ng/mL of IL-4 (PeproTech). Media was replenished on day 3. Non-adherent cells were collected and re-plated in complete RPMI-1640 with 20ng/mL of GM-CSF on day 6. α-GalCer (KRN7000; DiagnoCine) was sonicated for 20 min at 50°C before being added to the DC cultures at 0.4 µg/ml. DCs were collected the next day and 2 × 10^5^ cells were injected intravenously to induce NKT cell activation and proliferation.

### MTT assay

4T1 cells were infected at an MOI of 1 or 10 with VSV-GFP, VSV-p10ARV, VSV-p14, or VSV-p15. Media was removed 20 h post infection and 0.5 mg/ml MTT reagent (3-(4,5-dimethylthiazol-2-yl)-2,5-diphenyl tetrazolium bromide) (Sigma) diluted in PBS was added and incubated for 2 h at 37 °C. MTT reagent was removed and 100ul of DMSO was added for 20 min and incubated at 37 °C. Viability was examined on a plate reader (BMG Labtech) by subtracting the 690 nm absorbance value from the 540 nm absorbance value.

### Acid phosphatase assay

Viability of 3D spheroids was determined by acid phosphatase assay [42]. 4T1 and MCF7 cells were cultured for 6 days in mammosphere medium (DMEM/F-12 supplemented with 20 ng/mL basic fibroblast growth factor, 20 ng/mL epidermal growth factor, 100 U/mL penicillin, 100 µg/mL streptomycin, and 1× B27 serum-free supplement) to obtain spheroids. 4T1 and MCF7 spheroid cultures were plated at 1 × 10^4^ cells per well in 96 well plates. Cells were infected with 1 × 10^8^ PFU of VSV-GFP, VSV-p10ARV, VSV-p14, or VSV-p15 for 20 h. Spheroids were dissociated and resuspended in a 1:1 ratio of PBS and phosphatase solution (0.1 M sodium acetate, 0.1% Triton X-100, 4 mg/mL phosphatase substrate), and incubated for 90 min at 37 °C. The reaction was stopped by adding 50 µL of 1 N NaOH to each well and samples were centrifuged at 1,000 × g for 5 min. Supernatants were transferred to 96-well plates and absorbance was measured at 405 nm (BMG Labtech) to determine cytosolic acid phosphatase activity/viability.

### Infectivity assay

To measure viral production, 1.5 × 10^5^ 4T1 and MCF7 cells were plated in 12 well plates and infected at an MOI of 5 with VSV-GFP, VSV-p10ARV, VSV-p14, or VSV-p15 for 24 h. Supernatants were isolated and titre was determined by plaque assay on Vero cells.

### Immunogenic cell death

Tumors and blood were isolated 24 h after the final virus injection. Tumors were dissociated into a single cell suspension by mechanical dispersion. Tumor cells were then surface stained with anti-calreticulin (ab2907, Abcam) for 30 min followed by Alexflour647 secondary (A21244, Life technologies) for 30 min. Samples were read using a three-laser FACSCelesta and analysis was performed using FlowJo 10.7.1 software (BD Biosciences). Serum was isolated from blood using serum separator tubes (Sarstedt) and centrifuged at 3,500 x g for 20 min. Serum concentrations of HMGB1 and CXCL10 were determined by commercial ELISA kits (Elabscience and eBioscience, respectively).

### Generation of GCaMP6s 4T1 cell line

To generate the GCaMP6s 4T1 cell line, 4T1 cells were transduced with pLVX-GCaMP6s-Hygro lentivirus encoding the fluorescent calcium biosensor GCaMP6s [43]. Cells were plated at 1 × 10^5^ cells/well in 12 well plates and spin inoculated with lenti-pLVX-GCaMP6s-Hygro in serum-free DMEM for 1 h at 37 °C, and then incubated at 37 °C for 24 h in complete DMEM. Cells were then placed under hygromycin (100 µg/ml) selection for 72 h.

### Imaging and quantification of Ca2^+^ transients

To detect and quantify calcium transients, 2 × 10^4^ 4T1-GCaMP6s cells/well were plated in Nunc MicroWell 96-well optical bottom plates and incubated at 37 °C for 24 h. 4T1-GCaMP6s cells were infected with VSV-fluc or VSV-p15 at an MOI of 1 and cultured in 10% FBS FluoroBrite DMEM (Thermo Fisher) supplemented with 2mM L-glutamine, 10mM HEPES, 100 µg/mL streptomycin, and 100 units/mL penicillin. 4T1-GCaMP6s were illuminated with a 488 nm laser and images were captured every 0.45 s for 9 min at 20x magnification and 0.8 numerical aperture on a Zeiss Axios Observer Z.1 spinning disk confocal microscope with an AxioCam MR R3 camera. During acquisition, cells were maintained in a humified chamber at 5% CO_2_ and 37 °C. The Cellpose cell segmentation algorithm was used to automatically segment GCaMP6s expressing cells from an average intensity projection of the first 100 frames of the timelapse video [44]. Images were recorded using Zeiss Zen Blue software. Background fluorescence signal was subtracted using the ImageJ rolling ball algorithm [45], and F/F was calculated by dividing the mean fluorescence intensity of a cell at the time of acquisition by the cell mean intensity over the course of the time series.

### Flow cytometry and immune phenotyping

The following antibodies were obtained from BD Biosciences, eBioscience or BioLegend: fluorescein isothiocyanate-labelled TCRβ (clone H57-597) and Ly6C (clone HK1.4); Peridinin chlorophyll protein-Cyanine5.5-labelled CD49b (clone DX5) and F4/80 (clone BM8); Phycoerythrin-Cyanine7-labeled CD45 (clone 30-F11); Allophycocyanin-labelled CD80 (clone 16-10A1), and CD8α (clone 53 − 6.7); Allophycocyanin-eflour780-labelled CD11c (clone N418) and CD69 (clone H1.2F3); AlexaFloura700-labelled CD4 (clone RM4-5) and MHC II (clone M5/114.15.2); Brilliant violet (BV) 605-labelled CD8α (clone 53 − 6.7); BV650-labelled CD11b (clone M1/70); BV785-labelled PD-1 (29 F.1A12). Allophycocyanin-labeled CD1d tetramers loaded with the synthetic glycolipid PBS57 were obtained from the NIH Tetramer Core Facility (Emory University). Cells were incubated for 20 min at room temperature with eflour450 fixable viability dye (Thermo Fisher). Cells were washed and incubated 30 min at 4 °C with antibody panels in brilliant stain buffer (BD Biosciences) to stain cell subsets, washed, and fixed in 2% paraformaldehyde (Fisher Scientific). Samples were acquired using a three-laser FACSCelesta and analyzed with FlowJo 10.7.1 software (BD Biosciences).

Spleens and tumors were isolated and dispersed into single cell suspensions using mechanical dispersion through 70 μm wire mesh. Tumor infiltrating lymphocytes were enriched using a 30% Percoll gradient (Cytiva). Red blood cells were lysed using ammonium chloride buffer, followed by a wash with PBS containing 2% FBS. Lymphoid and myeloid populations were examined by flow cytometry.

### Clonogenic assay

Lung metastasis was quantified using an established clonogenic assay with 6-thioguanine selection for 4T1 cells [46]. Briefly, lungs were harvested from untreated and treated tumor-bearing mice on day 25, digested in a type IV collagenase/elastase cocktail and dissociated by mechanical dispersion through a sterile 40 micron nylon mesh. Total cells were resuspended in 2 ml of complete RPMI and cultured in dilution series with 60 µM 6-thioguanine (Alfa Aesar). After 10 days in culture, cell colonies were fixed with 1 ml 95% methanol for 5 min, washed twice with 1 ml of distilled water, and stained with 1 ml of 0.03% methylene blue (BioShop). The number of CFU were counted on each plate. The number of CFU in the whole lung tissue was calculated based on the number of colonies formed relative to the volume of total lung suspension.

### Cytotoxicity and cytokine production

Splenocytes were isolated and stained with allophycocyanin-labelled CD8α (clone 53 − 6.7) antibody. CD8α^+^ T cells, were isolated using EasySep allophycocyanin positive selection kit II (StemCell) and co-cultured at a 1:1 ratio with Oregon green-labelled 4T1 cells for 18 h in complete DMEM. After incubation, supernatant was collected to examine IFNγ and TNF levels by ELISA (eBioscience). Oregon green-labelled 4T1 cells were examined by flow cytometry using phycoerythrin-labeled annexin V (BioLegend) and 7-amino-actinomycin D (BioLegend) to identify apoptotic and dead cells.

### Statistics

Data are expressed as mean ± SEM unless otherwise stated. A non-parametric two-tailed Mann–Whitney U-test was used to compare between data groups. Survival data were analyzed by log-rank (Mantel–Cox) significance test. All results are representative of at least two independent biological repeats containing multiple mice. Significance was set at *P* < 0.05. Statistical computations were carried out using GraphPad Prism 8.4. Longitudinal analysis of tumor growth curves was performed by liner mixed-effect modeling followed by type II ANOVA and pairwise comparisons across groups using the online TumGrowth tool (https://kroemerlab.shinyapps.io/TumGrowth/) [47].

## Results

### VSV-FAST increase syncytia formation and 4T1 cell death in vitro

**Fig. 1:**
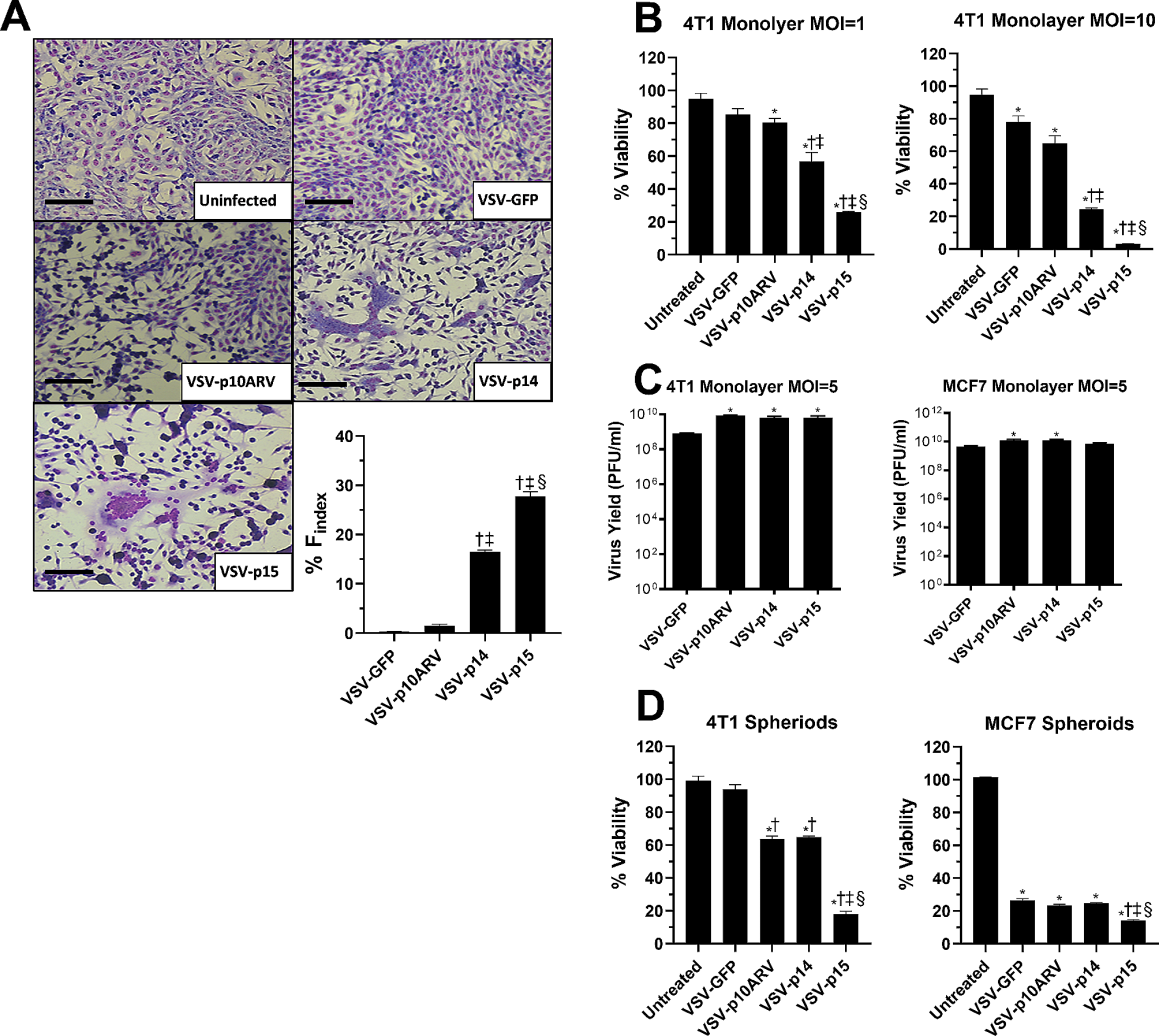
VSV-FAST increases 4T1 cell fusion and cell death *in vitro.* (**A**) 4T1 cell monolayers were cultured with vehicle (uninfected) or infected at an MOI of 1 with VSV-GFP, VSV-p10ARV, VSV-p14, or VSV-p15. Images of syncytia were acquired 12.5 h after infection. Scale bar is 100µM. The number of nuclei per syncytia was quantified at 15 h (*n* = 6 per group). ^†^*p* < 0.05 compared to VSV-GFP, ^‡^*p* < 0.05 compared to VSV-p10ARV, ^§^*p* < 0.05 compared to VSV-p14. (**B**) 4T1 cell monolayers were infected in culture for 20 h at an MOI of 1 or 10 with VSV-GFP or VSV expressing different FAST proteins. Viability was determined by MTT assay (*n* = 3 per group). ^*^p < 0.05 compared to untreated, ^†^*p* < 0.05 compared to VSV-GFP, ^‡^*p* < 0.05 compared to VSV-p10ARV, ^§^*p* < 0.05 compared to VSV-p14. (**C**) Monolayers of 4T1 cells or MCF7 cells were infected at an MOI of 5 for 24 h. Supernatants were collected and viral titres were determined by plaque assay (*n* = 3 per group). ^*^p < 0.05 compared to VSV-GFP. (**D**) Spheroids were collected from 4T1 and MCF7 cells infected with VSV-GFP or VSV expressing different FAST proteins for 20 h. Viability was determined by acid phosphatase assay (*n* = 3 per group). ^*^p < 0.05 compared to untreated, ^†^*p* < 0.05 compared to VSV-GFP, ^‡^*p* < 0.05 compared to VSV-p10ARV, ^§^*p* < 0.05 compared to VSV-p14

Reovirus FAST proteins p10ARV, p14, and p15 were cloned into the VSVΔM51 backbone and used to generate recombinant VSV as previously described [34]. To determine the effects of FAST proteins on VSV oncolytic activity, we infected 4T1 cells in vitro with VSV-GFP, VSV-p14, VSV-p15, or VSV-p10ARV at MOIs of 1–10 and examined syncytia formation, cell death, and viral load. 4T1 cells are highly metastatic and relatively resistant to VSV virotherapy, providing a good model to examine whether FAST proteins increase VSV killing of cancer cells [23, 34, 46, 48]. At low MOIs, VSV-GFP infection caused no syncytia, VSV-p10ARV induced small syncytia, VSV-p14 infection led to significant syncytium formation, while VSV-p15 was the most syncytiogenic (Fig. 1A). To examine the effect of FAST proteins on VSV killing activity against 4T1 cells, monolayers of 4T1 cells were infected at an MOI of 1 or 10 for 20 h and viability was examined using the MTT assay. At both MOIs, loss of viability correlated with syncytiogenic activity, with VSV-p15 inducing the greatest loss in viability followed by VSV-p14 and then VSV-p10 (Fig. 1B). Differences in killing activity of VSV-FAST constructs were not due to differing levels of virus replication since all three VSV-FAST viruses replicated to same the level in 4T1 cells, generating viral titers that were higher than those obtained from VSV-GFP-infected cells (Fig. 1B-C). Similarly, infection of 4T1 spheroids with VSV-p15 significantly increased cell death compared to VSV-GFP and the other VSV-FAST constructs (Fig. 1D). Similar findings were observed with spheroids of human MCF7 breast adenocarcinoma cells. While MCF7 cells were more susceptible to VSV-mediated killing, VSV-p15 infection significantly decreased cell viability compared to VSV-GFP, VSV-p10ARV, and VSV-p14, but did not exhibit increased replication compared to the other VSV constructs (Fig. 1C-D). These findings indicate that FAST proteins increase the oncolytic activity of VSV and enhanced oncolytic activity correlates with the syncytiogenic potential of the VSV-FAST construct.

### FAST proteins increase VSV oncolytic activity in vivo

**Fig. 2:**
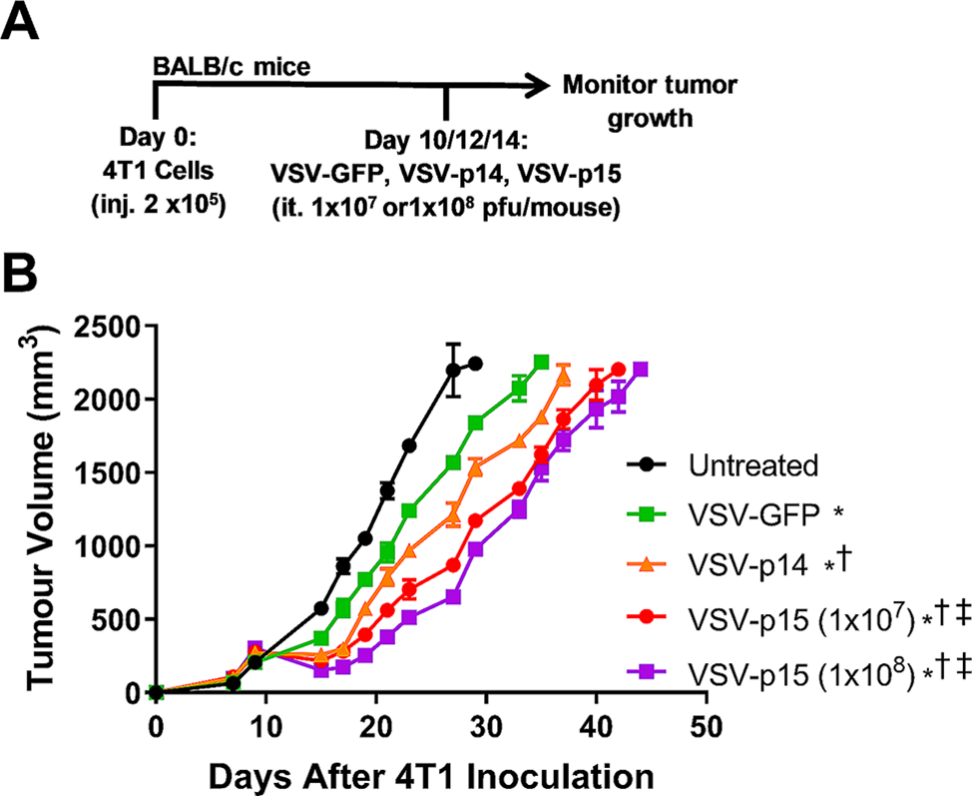
FAST proteins increase the anti-tumor activity of VSV in a primary 4T1 model. (**A**) Schematic of the primary 4T1 tumor model and treatment schedule. (**B**) 4T1 tumor volume was assessed in untreated tumor-bearing mice and mice treated with VSV-GFP, VSV-p14, VSV-p15 (1 × 10^7^), or VSV-p15 (1 × 10^8^) (*n* = 4–14 per group). Comparison of curves was performed via linear mixed-effect modeling analysis: ^*^p < 0.05 compared to untreated, ^†^*p* < 0.05 compared to VSV-GFP, ^‡^*p* < 0.05 compared to VSV-p14

We next examined whether FAST proteins could increase the anti-tumor activity of VSV in a primary 4T1 tumor model. Following implantation of 2 × 10^5^ 4T1 cells into the 4th mammary pad of female BALB/c mice, animals were injected intratumorally (it.) on days 10, 12, and 14 with 1 × 10^8^ PFUs of VSV-GFP or the fusogenic VSV-p10ARV, VSV-p14 or VSV-p15 constructs (Fig. 2 and Supplemental Fig. 1). VSV-GFP significantly slowed tumor progression compared to untreated mice, with VSV-p14 and VSV-p15 exhibiting further decreases in tumor progression relative to VSV-GFP treatment (Fig. 2B). Furthermore, the highly syncytiogenic VSV-p15 construct was more efficient than VSV-p14 at slowing tumor progression, even at a 10-fold lower dose of virus (Fig. 2B). VSV-p10ARV did not impair tumor growth any better than VSV-GFP (Supplemental Fig. 1), therefore VSV-p10ARV was dropped from further experiments due to lack of enhanced in vivo efficacy.

### FAST proteins increase VSV-induced immunogenic cell death

**Fig. 3:**
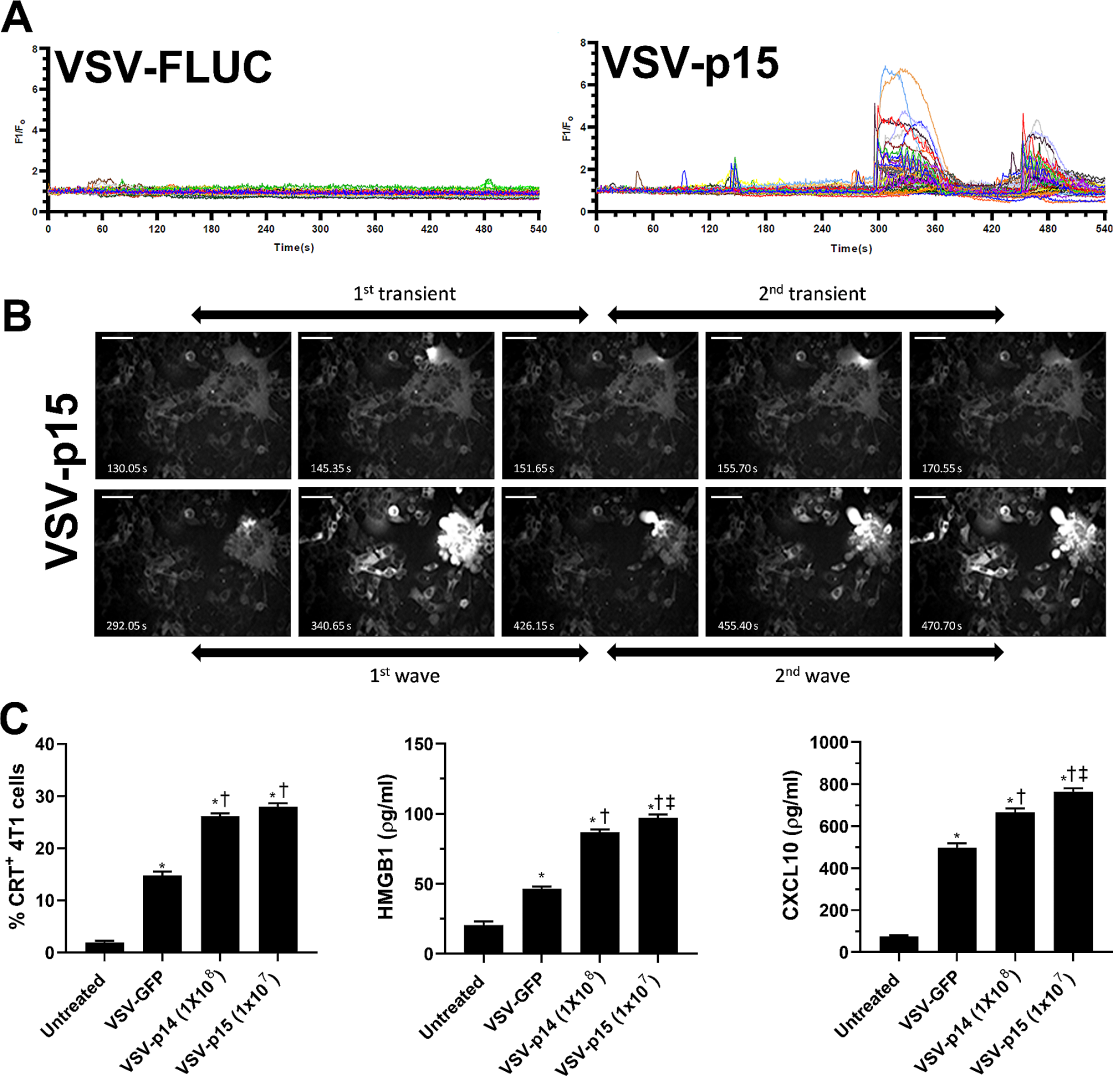
FAST proteins increase the VSV-induced markers of immunogenic cell death. Calcium flux reporter 4T1-GCaMP6s cells were infected with either VSV-fluc or VSV-p15 in vitro at an MOI of 1. (**A**) Single cell Ca^2+^ traces of 4T1-GCaMP6s cells 14 h post infection with either VSV-fluc or VSV-p15 were imaged at 2.22 Hz for 9 min. Each line represents a single cell (*n* = 40 per group). (**B**) A representative time course of Ca^2+^ fluxes in 4T1-GCaMP6s cells during syncytial death 14 h post VSV-p15 infection. Scale bar is 50µM. (**C**) Tumors and blood from untreated and treated tumor-bearing mice were harvested 24 h after final VSV treatment as performed in Fig. 2A. Tumors were isolated and dispersed into single cell suspensions. Flow cytometry was used to assess surface expression of calreticulin (CRT) (*n* = 8-9per group). HMGB1 and CXCL10 concentrations were determined in blood serum by ELISA (*n* = 3-9per group). ^*^p < 0.05 compared to untreated, ^†^*p* < 0.05 compared to VSV-GFP, ^‡^*p* < 0.05 compared to VSV-p14

An important mechanism underlying oncolytic virotherapy is the ability of oncolytic viruses to induce immunogenic cell death (ICD) and promote anti-tumor immune responses [49]. Previously, we demonstrated that VSV can induce ICD in 4T1 cells and other tumor lines [23]. Therefore, we examined whether the expression of FAST proteins could increase the ability of VSV to induce ICD. ER stress causes leakage of Ca^2+^ into the cytosol and is a major contributor to ICD [50, 51]. To examine the effects of FAST proteins on Ca^2+^ signalling, we transfected 4T1 cells with a genetically-encoded Ca^2+^ biosensor, GCaMP6s [43]. The following day, cells were infected with either VSV-p15 or VSV expressing firefly luciferase (VSV-fluc) at an MOI 1; VSV-fluc was used as a control instead of VSV-GFP, since GFP emits at the same wavelength as GCaMP6s. Live cells were illuminated with a 488 nm laser starting at 14 h post-infection when syncytia were apparent, and images were captured every 0.45 s for 9 min. As shown in Supplemental video 1 and fluorescence quantification (Fig. 3A), VSV-Fluc caused only minor fluctuations in GCaMP fluorescence over time. In marked contrast, VSV-p15 induced small Ca^2+^ transients in syncytia, followed shortly thereafter by intense fluorescence peaks in the syncytia as they contracted and died. These intense Ca^2+^ spikes rapidly emanated out in waves of decreasing amplitude through adjacent cells (Fig. 3B and Supplemental video 1). These results are consistent with a previous study linking Ca^2+^ signalling to FAST protein-induced syncytium formation [52] and provide one readout suggesting that FAST protein-induced syncytia undergo ICD. Moreover, the paracrine effect of disrupted Ca^2+^ signalling in syncytia on adjacent cells suggests effects may be more extensive than just in syncytia. Paracrine signals such as the interaction of ADP released from syncytia with purinergic receptors on neighbouring cells may explain this effect, as recently shown for the NSP4 viroporin in rotavirus-infected cells [53].

To confirm that FAST proteins expressed by VSV can enhance ICD, other key markers of ICD were examined 24 h after the third virus injection into primary 4T1 tumors. Cytosolic Ca^2+^ signalling induces surface expression of calreticulin (CRT), an endoplasmic reticulum chaperone whose surface expression on dying tumor cells triggers uptake by antigen presenting cells and contributes to ICD [54, 55]. Flow cytometry of single cell tumor suspensions revealed VSV-p14 at a dose of 1 × 10^8^ PFU and VSV-p15 at 1 × 10^7^ PFU both significantly increased surface expression of CRT compared to VSV-GFP (Fig. 3C). ICD is also associated with release of HMGB1, which binds to RAGE to enhance DC maturation and homing to lymph nodes [56], and also binds TLR2 and 4 leading to increased inflammatory cytokine production from DCs [56]. Recent studies also identified CXCR3 ligands, such as CXCL10, as another critical component of ICD [57]. Therefore, we examined serum levels of HMGB1 and CXCL10. Consistent with reports that oncolytic VSV induces ICD [23, 58], VSV-GFP increased serum levels of HMGB1 and CXCL10 compared to untreated mice (Fig. 3C). However, VSV-p14 and VSV-p15 (at a 10-fold lower dose) both significantly increased serum levels of HMGB1 and CXCL10 compared to VSV-GFP-treated mice, indicating that FAST proteins increase the ability of VSV to induce ICD.

### Oncolytic VSV-FAST increases immune cell infiltration and activation

**Fig. 4:**
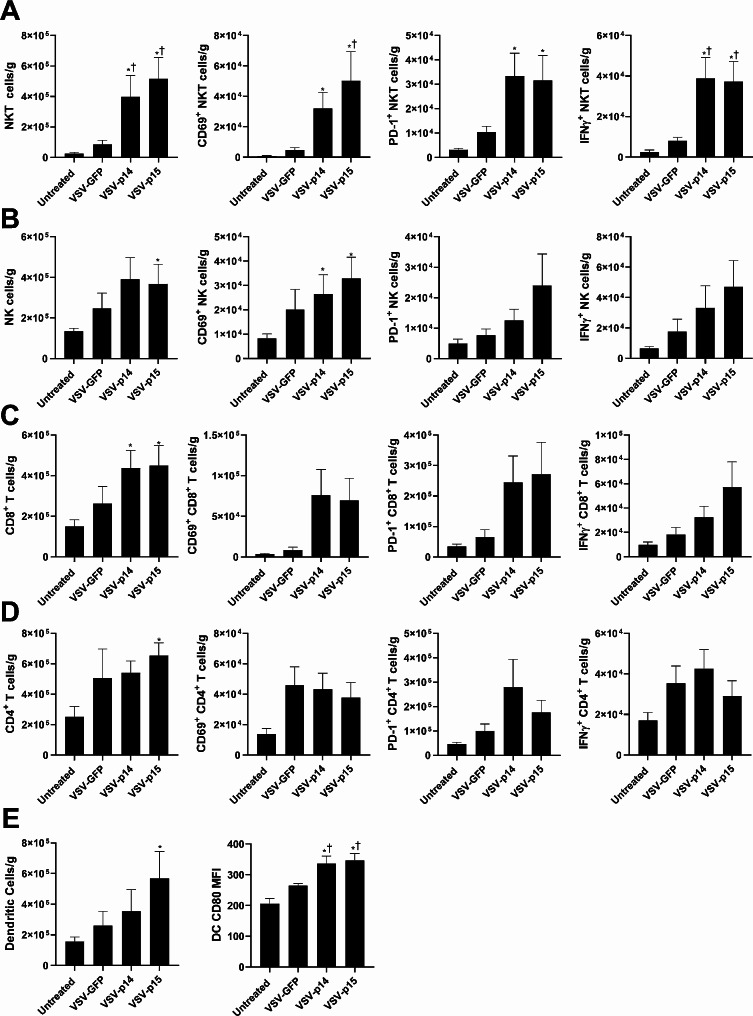
VSV-FAST constructs increase immune infiltration and activation in 4T1 tumors. Mice were treated as described in Fig. 2A. Twenty-four hours after the final virus treatment, tumors were isolated and dispersed into single cell suspensions. (**A**-**B**) Flow cytometry was used to assess immune cell infiltration (*n* = 8–9 per group). The number of **(A)** NKT cells (CD1d tetramer^+^ TCRβ^+^), **(B)** NK cells (NK1.1^+^ TCRβ^−^) (**C**) CD8^+^ T cells (TCRβ^+^ CD8α^+^), (**D**) CD4^+^ T cells (TCRβ^+^ CD4^+^) and the expression of CD69, PD-1, and intracellular IFNγ by these subsets was assessed. (**E**) The number of dendritic cells (MHC II^+^ CD11c^+^) and CD80 expression was also examined.^*^p < 0.05 compared to untreated, ^†^*p* < 0.05 compared to VSV-GFP

Oncolytic viruses work in part by increasing immune cell infiltration into tumors [27, 28]. To examine whether FAST proteins affect immune infiltration and anti-tumor activity, we examined immune populations in the spleen and tumor 24 h after the final virus treatment. VSV-p14 and VSV-p15 both significantly increased the number of tumor infiltrating NKT cells compared to VSV-GFP (Fig. 4A). These cells exhibited markers associated with enhanced activation, including surface expression of CD69, PD-1, and intracellular production of IFNγ (Fig. 4A). VSV-p15 also significantly increased NK cell tumor infiltration (Fig. 4B), and both VSV-p14 and VSV-p15 increased NK cell CD69 expression, demonstrating increased activation by VSV expressing FAST proteins. While VSV-p14 significantly increased tumor infiltration of CD8 T cells (Fig. 4C), VSV-p15 increased tumor infiltration of CD8 T cells (Fig. 4C) and CD4 T cells (Fig. 4D). Activation markers on CD8 and CD4 T cells trended toward increased expression but did not reach statistical significance (Fig. 4C-D). VSV-p15 enhanced infiltration of DCs compared to other treatments (Fig. 4E), but both VSV-p14 and VSV-p15 significantly increased CD80 expression on tumor infiltrating DCs, demonstrating an increase in DC activation. These results in tumors were similar to those observed in the spleen, where VSV-p14 and VSV-p15 tended to increase the numbers and activation state of NKT cells (increased CD69, PD-1, and IFNγ expression) and DCs (increased CD80 expression) (Supplemental Fig. 2A-E). Taken together, VSV-p14 and VSV-p15 enhance the immune response when compared to VSV-GFP treatments and untreated mice.

### VSV-FAST in combination with NKT cell immunotherapy controls 4T1 metastatic disease

**Fig. 5:**
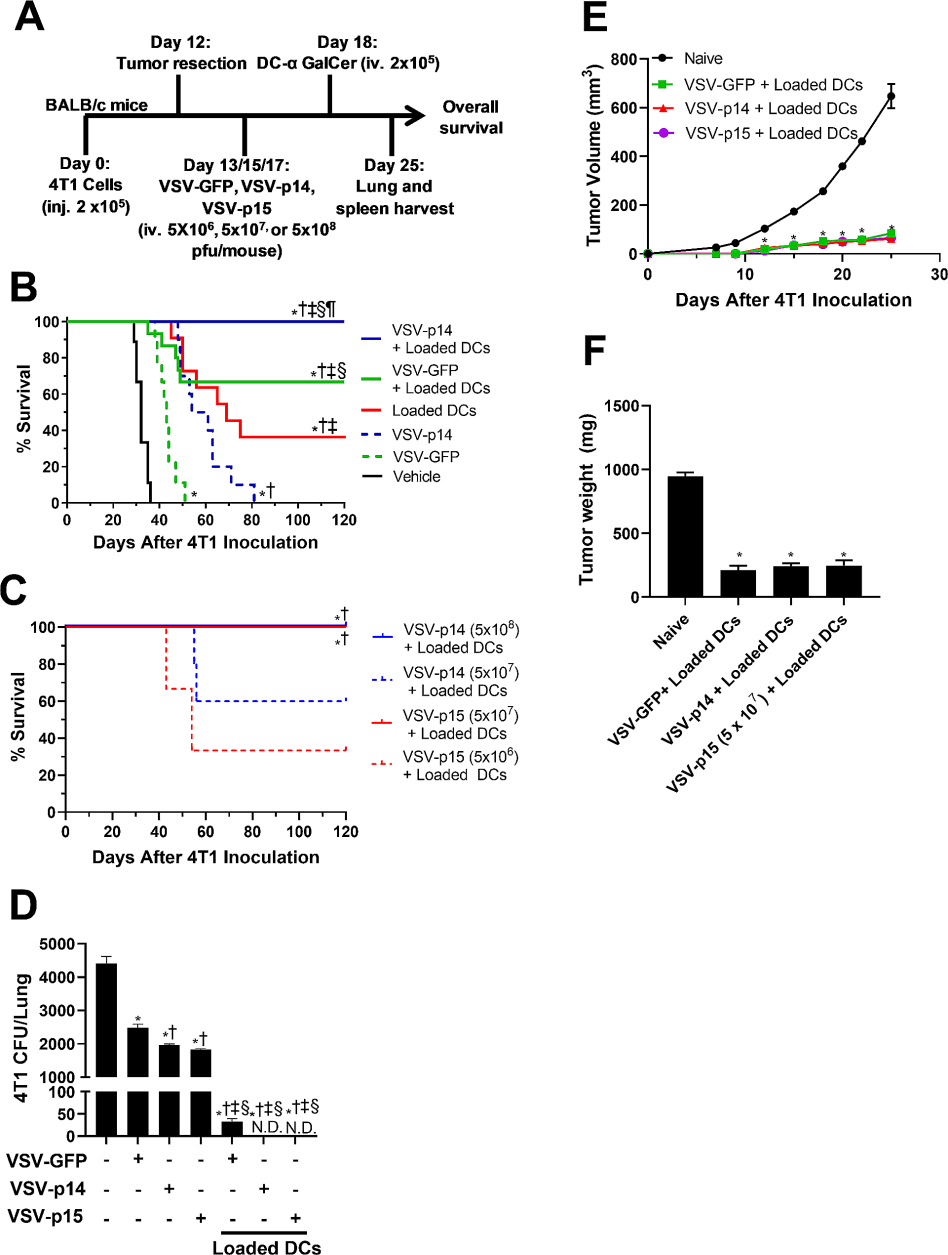
Combination therapy controls lung metastasis in a metastatic breast cancer model. (**A**) Schematic of the metastatic 4T1 tumor model and treatment schedule. (**B**) Overall survival was assessed in untreated tumor-bearing mice and mice treated with VSV-GFP or VSV-p14, alone and in combination with NKT cell immunotherapy mediated by transfer of α-GalCer-loaded DCs (*n* = 9–14 per group). ^*^p < 0.05 compared to untreated, ^†^*p* < 0.05 compared to VSV-GFP, ^‡^*p* < 0.05 compared to VSV-p14, ^§^*p* < 0.05 compared to Loaded DCs. ^¶^*p* < 0.05 compared to VSV-GFP + glycolipid-loaded DCs. (**C**) Overall survival was assessed in mice receiving different doses of VSV-p14 or VSV-p15 in combination with NKT cell immunotherapy (*n* = 3–8 per group). ^*^p < 0.05 compared to VSV-p15 (5 × 10^6^) + glycolipid-loaded DCs, ^†^*p* < 0.05 compared to VSV-p14 (5 × 10^7^) + glycolipid-loaded DCs. (**D**) Lungs were isolated and dispersed into single cell suspensions to assess metastasis of 4T1 cells by colony-forming assay in the presence of 6-thioguanine (*n* = 4–7 per group). ^*^p < 0.05 compared to untreated, ^†^*p* < 0.05 compared to VSV-GFP, ^‡^*p* < 0.05 compared to VSV-p14, ^§^*p* < 0.05 compared to VSV-p15. (**E**, **F**) Treated mice that survived to day 120 (Fig. 5B, C) were re-challenged in the contralateral mammary fat pad with 4T1 cells. Tumor (**E**) volume and (**F**) tumour weight were compared to naïve mice inoculated with 4T1 cells. *p < 0.05 compared to naïve control

Surgical resection is the most effective way of treating primary breast tumors, however, we currently lack effective approaches to cure metastatic breast cancer. Earlier work has shown that VSV can increase the therapeutic benefit of NKT cell immunotherapy in a breast cancer metastasis model [23]. To determine whether VSV-p14 and VSV-p15 can further increase the efficacy of NKT cell immunotherapy, 2 × 10^5^ 4T1 cells were injected into the 4th mammary pad of female BALB/c mice and tumors were surgically resected on day 12, when micro-metastases have already been seeded [46]. Mice were treated with VSV-GFP or VSV-p14 (iv. 5 × 10^8^ PFUs), or with VSV-p15 (iv. 5 × 10^7^ PFUs) on days 13, 15, and 17, followed by unloaded (control) or α-GalCer loaded DCs (iv. 2 × 10^5^) on day 18 to activate NKT cells (Fig. 5A). When used as monotherapies, VSV-GFP and VSV-p14 increased overall survival time compared to untreated mice, with VSV-p14 being more effective than VSV-GFP (Fig. 5B). Consistent with what we have shown previously, NKT cell activation with glycolipid-loaded DCs resulted in ∼ 40% survival at experimental endpoint, while ∼ 70% of mice receiving combined treatment with VSV-GFP plus glycolipid-loaded DCs survived (Fig. 5B) [13, 23]. Strikingly, 100% of mice treated with VSV-p14 and glycolipid-loaded DCs survived to endpoint (Fig. 5B). To examine whether the increased efficacy of VSV-p15 in the primary tumor model (Fig. 2) also carried over to the metastatic breast cancer model, VSV-p14 and VSV-p15 were tested at different doses in combination with NKT cell immunotherapy. VSV-p15 mediated 100% survival at a dose of 5 × 10^7^ PFU (Fig. 5C), a 10-fold lower dose than that required for VSV-p14 to mediate protection; treatment with 5 × 10^7^ PFU VSV-p14 only mediated survival in ∼ 60% of mice (Fig. 5C). A further 10-fold decrease in the VSV-p15 dose to 5 × 10^6^ PFU also decreased efficacy with a survival rate of ∼ 30%. Thus, VSV expressing p14 or p15 FAST proteins worked in combination with NKT cell immunotherapy to provide 100% protection against breast cancer metastases, with VSV-p15 being effective at a 10-fold lower dose.

Mice treated systemically with VSV-p14 or VSV-p15 exhibited transient flu-like symptoms that resolved rapidly. While p14 expressed in the parental unattenuated VSV strain causes increased neuropathogenesis and paralysis in mice [59], we did not observe any signs of paralysis or altered behavior with our VSVΔM51 constructs expressing p10ARV, p14 or p15. This is consistent with previous reports that VSVΔM51-p14 constructs do not cause paralysis and are cleared from the brain at the same rate as VSV-GFP [34, 59].

Lungs were isolated from mice on day 25 to examine the effects of our treatments on metastatic burden via an established clonogenic plating assay [46]. VSV-GFP treatment on its own decreased metastatic burden by ∼ 50% compared to untreated mice, with VSV-p14 and VSV-p15 further decreasing lung metastatic burden compared to VSV-GFP (Fig. 5D), consistent with the metastatic survival results (Fig. 5B-C). When used in combination with NKT cell immunotherapy, VSV-GFP dramatically reduced metastatic burden while VSV-p14 and VSV-p15 (at 1/10th the dose) reduced metastatic burden to undetectable levels (Fig. 5D).

To examine whether mice who survived metastatic disease to day 120 would exhibit immune memory and resist subsequent tumor rechallenge, mice were inoculated with 2 × 10^5^ 4T1 cells into the contralateral mammary fat pad. Mice that had been treated with VSV-GFP, VSV-p14, or VSV-p15 in combination with NKT cell immunotherapy all exhibited impaired tumor growth (Fig. 5E) and reduced endpoint tumor weight (Fig. 5F) compared to naïve mice, demonstrating a memory immune response against the tumor cells. There was no difference in tumor growth or weight between the different treatment groups, indicating all surviving mice had established a strong memory response.

### VSV-FAST in combination with NKT cell immunotherapy increases immune function

**Fig. 6:**
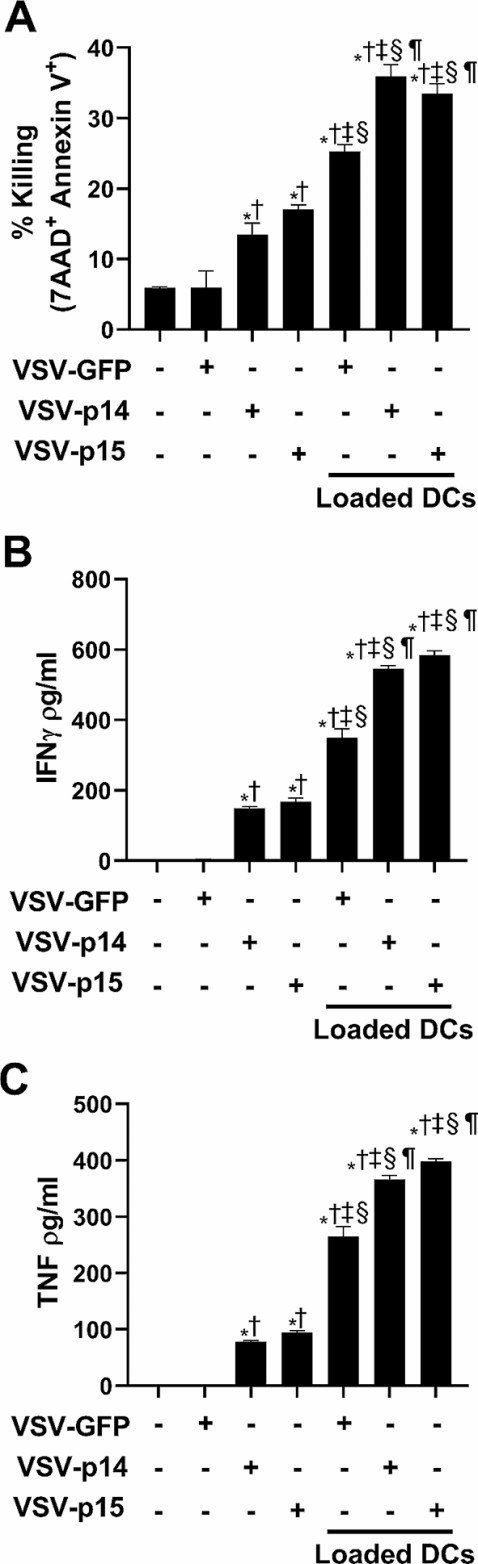
Combination therapy increases CD8 T cell cytotoxic activity and pro-inflammatory cytokine production. Spleens from untreated tumor-bearing and treated mice were isolated seven days after treatment as outlined in Fig. 5A and dispersed into single cell suspensions. (**A**) CD8^+^ T cells were isolated by magnetic bead purification and cocultured 1:1 with Oregon green-labelled 4T1 cells. Cells were stained with Annexin V and 7AAD after 18-hour to determine cytotoxic activity against 4T1 cells. (**B**, **C**) Supernatants were collected from the 18-hour cocultures to measure cytokine production. Concentrations of (**B**) IFNγ and (**C**) TNF were determined by ELISA (*n* = 3 per group). ^*^p < 0.05 compared to untreated, ^†^*p* < 0.05 compared to VSV-GFP, ^‡^*p* < 0.05 compared to VSV-p14, ^§^*p* < 0.05 compared to VSV-p15, ^¶^*p* < 0.05 compared to VSV-GFP + glycolipid-loaded DCs

To examine the antigen-specific immune response against 4T1, splenic CD8^+^ T cells were sorted from untreated and treated mice and cocultured with 4T1 cells for 18 h. Cytotoxicity against 4T1 cells was assessed by 7AAD and Annexin V staining and cytokine release was assessed by ELISA. CD8^+^ T cells from mice that received VSV-p14 or VSV-p15 treatment alone exhibited increased cytotoxicity against 4T1 cells relative to untreated and VSV-GFP treated mice (Fig. 6A). When virotherapy was combined with NKT cell immunotherapy, treatments resulted in significantly increased CD8 + T cell cytotoxicity, with VSV-p14 and VSV-p15 significantly increasing 4T1 cell killing compared to VSV-GFP (Fig. 6A). Similarly, cocultures using CD8 + T cells from mice that received VSV-FAST viruses alone or in combination with immunotherapy exhibited increases in the release of the proinflammatory anti-cancer cytokines IFNγ (Fig. 6B) and TNF (Fig. 6C) into the culture supernatant. Increased antigen-specific responses induced by combined treatments are consistent with impaired tumor growth in rechallenged mice that survived the first tumor challenge (Fig. 5E-F). Together, these data indicate that VSV-FAST viruses induced tumor-specific immune responses that were greatly enhanced in combination with NKT cell activation.

**Fig. 7:**
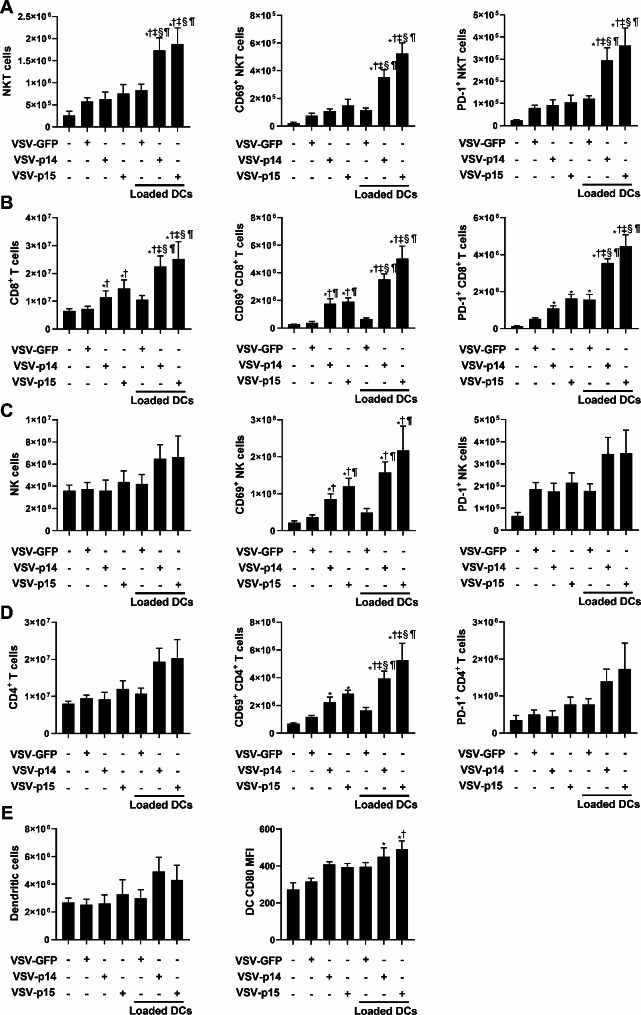
VSV-p14 or VSV-p15 in combination with NKT cell activation increases immune activation. VSV and NKT cell immunotherapy treatments were administered in the metastatic 4T1 model as outlined in Fig. 5A. Spleens from untreated and treated mice were isolated seven days after treatment. Flow cytometry was used to assess immune cell expansion and activation (*n* = 5–9 per group). The number of (**A**) NKT cells (CD1d tetramer^+^ TCRβ^+^), (**B**) NK cells (NK1.1^+^ TCRβ^−^) (**C**) CD8^+^ T cells (TCRβ^+^ CD8α^+^), (**D**) CD4^+^ T cells (TCRβ^+^ CD4^+^) and the expression of CD69, PD-1, and intracellular IFNγ by these subsets was assessed. (**E**) The number of dendritic cells (MHC II^+^ CD11c^+^) and CD80 expression were also examined. ^*^p < 0.05 compared to untreated, ^†^*p* < 0.05 compared to VSV-GFP, ^‡^*p* < 0.05 compared to VSV-p14, ^§^*p* < 0.05 compared to VSV-p15, ^¶^*p* < 0.05 compared to VSV-GFP + glycolipid-loaded DCs

To further examine the immune response, cell populations in the spleen of untreated and treated mice were examined. Spleens from mice treated with VSV-p14 or VSV-p15 in combination with NKT cell activation exhibited significant increases in NKT and CD8^+^ T cells compared to virus treatment alone or combination therapy with VSV-GFP (Fig. 7A-B). Furthermore, NKT and CD8^+^ T cells from animals treated with VSV-p14 or VSV-p15 in combination with NKT cell activation had increased expression of CD69 and PD-1 (Fig. 7A-B), demonstrating increased immune cell activation. While numbers of splenic NK cells and CD4 T cells were not significantly altered by treatments (Fig. 7C-D), VSV-p14 or VSV-p15 alone increased CD69 expression on NK cells and CD4^+^ T cells, with combination treatment increasing expression further. CD69 expression on NK cells and CD4^+^ T cells did not increase in mice treated with VSV-GFP alone or in combination with NKT cell activation, suggesting the increase was due to FAST proteins (Fig. 7C-D). While none of the treatments increased the number of DCs in the spleen (Fig. 7E), NKT cell activation in combination with VSV-p14 or VSV-p15 increased CD80 expression on the DCs, indicating increased maturation/activation. Taken together, therapy using VSV-p14 or VSV-p15 in combination with NKT cell activation significantly increased immune activation and function leading to reductions in lung metastases and increased survival.

## Discussion

Previously our laboratory has shown that oncolytic VSV and NKT cell immunotherapy can be effectively combined to increase survival in a metastatic 4T1 breast cancer model [23]. However, combination treatment only led to ∼ 70% survival, leaving room for therapeutic improvements. Here we demonstrate that NKT cell immunotherapy combined with oncolytic VSV expressing the FAST proteins p14 or p15 enhances control of metastatic burden and leads to 100% survival.

Fusogenic VSV constructs expressing FAST proteins all exhibited enhanced replication in vitro compared to VSV-GFP, but had differential killing ability, with VSV-p14 and especially VSV-p15 mediating the greatest loss in tumor cell viability. Previous results from our group indicate that increased viral replication may not translate in vivo, where VSV-p14 was cleared as quickly as VSV-GFP [34]. This suggests that other features mediated by the FAST proteins are critical for enhancing tumor control. Tumor killing activity in vitro correlated with the syncytiogenic activity of VSV-FAST constructs. As syncytia formation has previously been shown to increase oncolytic activity [60–62], this likely contributes to increased in vitro 4T1 cell killing with VSV-p14 and VSV-p15 compared to VSV-p10ARV and VSV-GFP. Enhanced syncytia formation and tumor cell killing, along with increased release of immunogenic cell death markers and tumor antigens, would be consistent with the ability of VSV-p14 and VSV-p15 to enhance anti-tumor immunity and metastatic control in vivo. Whether other related membrane events, such as increased release of tumor antigens in extracellular vesicles [61], also contribute to the protection mediated by VSV-FAST constructs requires further investigation. Intriguingly, VSV-p15 was the most effective FAST construct at inducing syncytia and tumor cell killing and was able to mediate tumor control in vivo at a 10-fold lower dose than VSV-p14.

While p15 and p14 both come from *Orthoreoviruses*, there is little sequence conservation between them, and recent analysis suggests these two FAST proteins may have evolved independently [63]. These proteins also have distinct structural and functional motifs, some of which are interchangeable, but some are not [64, 65]. For example, p14 and p15 both contain myristoylated N-terminal fusion peptides, but they are structurally and functionally distinct [38, 60, 66, 67]. Similarly, both contain membrane-proximal amphipathic helices in their cytoplasmic domains that are positioned to insert into the curved rim of a nascent fusion pore, lowering the energy barrier for pore formation [35], but the p14 and p15 motifs are structurally distinct and the p15 motif functions more efficiently than p14 to induce syncytium formation. These and other unique attributes of different FAST proteins likely contribute to their differences in syncytia formation, ICD, and in vivo tumor control.

Although oncolytic viruses can kill tumor cells, they likely work primarily by stimulating anti-tumor immunity and increasing immune infiltration to make tumors immunologically “hotter”. Consistent with this, intact viruses with limited replicative capacity can mediate efficient tumor control [68–70]. The increase in tumor infiltrating immune cells makes subsequent immunotherapies more effective [27–29, 71]. VSV-p14 and VSV-p15 increased NKT, NK, CD4 and CD8 T cell infiltration into 4T1 tumors and increased expression of activation (CD69) and effector (IFNγ) molecules. This sets the stage for oncolytic viruses to increase the efficacy of other immunotherapies.

VSV has been shown to stimulate antitumor immunity by causing ICD of cancer cells [49]. FAST proteins cause ER stress that can disrupt Ca^2+^ homeostasis, a major factor in ICD, indicating they may increase ICD [52, 54]. Additionally, the ER stress induced by FAST proteins activates the protein kinase RNA-like ER kinase (PERK) pathway, which is known to induce ICD [51, 72]. Syncytia formation has been associated with increased tumor antigen release, ICD, and stronger anti-tumor immune responses, with fusogenic viruses mediating increased anti-tumor immune responses compared to their non-fusogenic controls [60–62, 73]. An important mediator of ICD is surface mobilization of CRT, which functions as an “eat me” signal and enhances uptake of tumor antigens into antigen presenting cells [54]. VSV-p14 and VSV-p15 infection increased CRT surface expression more than VSV-GFP infection. However, VSV-p15 increased CRT at a 10-fold lower dose, indicating that VSV-p15 increases CRT more effectively than VSV-p14.

Other important mediators associated with ICD are extracellular release of HMGB1 and CXCL10. VSV-p15 significantly increased HMGB1 release compared to VSV-p14 and VSV-GFP. HMGB1 is an important mediator for migration and maturation of antigen presenting cells including DCs [56, 74]. Consistent with this, combination treatment of NKT cell immunotherapy with VSV-p14 or VSV-p15 increased the number of splenic and tumor infiltrating CD80^+^ DCs in both our primary and metastatic models. VSV-p15 also significantly increased extracellular CXCL10 compared to VSV-p14 and VSV-GFP. CXCL10 is a potent chemoattractant for lymphocytes including NKT cells [75], increasing immune cell tumor infiltration and immunotherapy efficacy [71]. Taken together, VSV-p15 infection increased ICD and immune cell infiltration more than VSV-p14 and VSV-GFP, even when used at a 10-fold lower dose, potentially due to its increased ability to form syncytia [60–62].

Previous studies have shown that that NKT cell activation by α-GalCer induces CD4 and CD8 memory T cell formation [13, 76]. These responses appear to be tumor specific as we found that following NKT cell activation, CD8 T cells isolated from mice harbouring Panc02 pancreatic adenocarcinoma tumors were able to recognize Panc02 cells in vitro but not B16 melanoma cells [29]. In contrast, NKT cell activation appears to cause non-selective priming of NK cells as they exhibited enhanced responses against both tumor lines. Consistent with our observation of increased CD8 T cell recognition of 4T1 cells in the current study, is plausible that NKT cell activation synergises with the increased immunogenic cell death and tumor antigen release induced by the VSV-FAST constructs to boost immunity. Importantly, this immune memory may help limit breast cancer recurrence as we found that growth of a 4T1 tumors in surviving mice receiving a second tumor challenge was impaired. Triple negative breast cancer has the highest recurrence rate of any breast cancer, occurring in 33.9% of treated patients within 2.6 years [77]. It is possible that the immune memory formed after our combination treatment could reduce the number of recurrences or extend the time until recurrence if applied to patients.

Overall, our current combined treatment of VSV-FAST constructs with NKT cell immunotherapy presents an effective and novel approach to treating breast cancer metastasis. The current standards of care are often ineffective and come with many severe adverse events [5, 6]. In contrast, VSV infection is associated with transient flu-like symptoms [78], and NKT cell activation is associated mainly with low level adverse effects [79]. The addition of FAST proteins to the VSVΔM51 construct does not mediate enhanced neuropathology associated with the parental strain [59], or enhance the persistence in non-tumor tissues [34]. Additionally, the reduced therapeutic titre of VSV-p15 needed for treatment may reduce adverse effects compared to combination treatment with delivery of higher dose VSV-GFP or VSV-p14. The decreased therapeutic viral titre of VSV-p15 may also reduce manufacturing and treatment costs and logistics, making the treatment more accessible.

In addition to the lungs, the 4T1 model metastasizes to multiple other sites including lymph nodes, liver, brain, and bone [46]. While we did not examine metastatic burden at these sites, we expect our therapy targeted metastatic burden at these locations as VSV and DCs were delivered systemically, and we observed no late morbidity in our model. It is also likely that our combined therapeutic approach will be effective in other cancers. We have previously shown that NKT cell immunotherapy combined with VSV-GFP enhanced survival in an ID8 ovarian cancer model [13], and VSV expressing the cytokine IL-15 could combine with NKT cell immunotherapy to reduce growth of Panc02 pancreatic adenocarcinoma tumors [29]. Furthermore, VSV-p14 also reduced metastasis of CT26 colon carcinoma cells to the lung [34]. Therefore, our therapeutic approaches with VSV-FAST constructs and NKT cell activation have the potential to improve patient outcomes for metastatic breast cancer and other cancers.

### Electronic supplementary material

Below is the link to the electronic supplementary material.

Supplementary Material 1: **Sup. Fig 1: p10ARV does not increase activity of VSV and overall survival in a primary 4T1 model.** 4T1 tumor volume was assessed in untreated tumor-bearing mice and mice treated with10^8^PFU VSV-GFP or VSV-p10ARV administered following the same timeline as Fig 2A (n= 10-14 per group). Untreated and VSV-GFP data are shown from figure 2B.

Supplementary Material 2: **Sup. Figure 2: VSV-FAST constructs increase immune activation in the spleen in a primary 4T1 tumor model.** Spleens from untreated tumor-bearing mice and treated mice were isolated and dispersed into single cell suspensions. Flow cytometry was used to assess immune cellexpansion and activation in the spleen seven days after the end of treatment (n= 8-9 per group). The number of A) NKT cells (CD1d tetramer^+^ TCRβ^+^), B) NK cells (NK1.1^+^ TCRβ^-^) C) CD8^+^ T cells (TCRβ^+^ CD8α^+^), D) CD4^+^ T cells (TCRβ^+^ CD4^+^) and the expression of CD69, PD-1, and intracellular IFNγ by these subsets was assessed. E) The number of dendritic cells (MHC II^+^CD11c^+^) and CD80 expression was also examined. ^*^p<0.05compared to untreated, ^†^P<0.05 compared to VSV-GFP.

Supplementary Material 3: **Supplemental video 1:**  VSV-p15mediates calcium flux during syncytial cell death. Ca2^+^ flux in VSV-p15 infected 4T1-GCaMP6s cells was recorded during syncytial death. A 3-dimensional gaussian blur has been applied to denoise the video.

## Data Availability

Requests for resources and reagents should be directed to and will be fulfilled by the Lead Contact, Dr. Brent Johnston (brent.johnston@dal.ca).

## Abbreviations

α-GalCer: α-galactosylceramide
CFU: Colong forming units
CRT: Calreticulin
DCs: Dendritic cells
FAST: Fusion associated small transmembrane
Fluc: Firefly luciferase
HMGB1: High mobility group box 1
ICD: Immunogenic cell death
It: Intratumoral
Iv: Intravenous
NK cell: Natural killer cell
NKT cell: Natural killer T cell
PFU: plaque forming units
VSV: Vesicular stomatitis virus

## References

[1] Siegel RL, Miller KD, Jemal A, Cancer statistics, 2019. CA Cancer J Clin 2019;69:7–34. 10.3322/caac.21551 10.3322/caac.21551 30620402

[2] Wahba HA, El-Hadaad HA (2015). Current approaches in treatment of triple-negative breast cancer. Cancer Biol Med.

[3] Anders CK, Carey LA (2009). Biology, metastatic patterns, and treatment of patients with triple-negative breast cancer. Clin Breast Cancer.

[4] Steeg PS (2016). Targeting metastasis. Nat Rev Cancer.

[5] Wysocki PJ, Korski K, Lamperska K, Zaluski J, Mackiewicz A. Primary resistance to docetaxel-based chemotherapy in metastatic breast cancer patients correlates with a high frequency of BRCA1 mutations. Med Sci Monit. 2008;14:SC7–10. http://www.medscimonit.com/abstract/index/idArt/863649 18591931

[6] Hryciuk B, Szymanowski B, Romanowska A, Salt E, Wasąg B, Grala B (2018). Severe acute toxicity following gemcitabine administration: a report of four cases with cytidine deaminase polymorphisms evaluation. Oncol Lett.

[7] Luqmani YA (2005). Mechanisms of drug resistance in cancer chemotherapy. Med Princ Pract.

[8] Fujii S-I, Shimizu K (2019). Immune networks and therapeutic targeting of iNKT cells in cancer. Trends Immunol.

[9] McEwen-Smith RM, Salio M, Cerundolo V (2015). The regulatory role of invariant NKT cells in tumor immunity. Cancer Immunol Res.

[10] Nelson A, Lukacs JD, Johnston B (2021). The current landscape of NKT cell immunotherapy and the hills ahead. Cancers (Basel).

[11] Benlagha K, Weiss A, Beavis A, Teyton L, Bendelac A (2000). In vivo identification of glycolipid antigen-specific T cells using fluorescent CD1d tetramers. J Exp Med.

[12] Fujii S, Shimizu K, Kronenberg M, Steinman RM (2002). Prolonged IFN-[gamma]-producing NKT response induced with [alpha]-galactosylceramide-loaded DCs. Nat Immunol.

[13] Gebremeskel S, Clattenburg DR, Slauenwhite D, Lobert L, Johnston B (2015). Natural killer T cell activation overcomes immunosuppression to enhance clearance of postsurgical breast cancer metastasis in mice. Oncoimmunology.

[14] Toura I, Kawano T, Akutsu Y, Nakayama T, Ochiai T, Taniguchi M (1999). Cutting edge: inhibition of experimental tumor metastasis by dendritic cells pulsed with α-galactosylceramide. J Immunol.

[15] Metelitsa LS, Naidenko OV, Kant A, Wu H-W, Loza MJ, Perussia B (2001). Human NKT cells mediate antitumor cytotoxicity directly by recognizing target cell CD1d with bound ligand or indirectly by producing IL-2 to activate NK cells. J Immunol.

[16] Cullen R, Germanov E, Shimaoka T, Johnston B (2009). Enhanced tumor metastasis in response to blockade of the chemokine receptor CXCR6 is overcome by NKT cell activation. J Immunol.

[17] Coquet JM, Chakravarti S, Kyparissoudis K, McNab FW, Pitt LA, McKenzie BS (2008). Diverse cytokine production by NKT cell subsets and identification of an IL-17–producing CD4– NK1.1– NKT cell population. Proc Natl Acad Sci.

[18] Motohashi S, Okamoto Y, Yoshino I, Nakayama T (2011). Anti-tumor immune responses induced by iNKT cell-based immunotherapy for lung cancer and head and neck cancer. Clin Immunol.

[19] Metelitsa LS, Wu H-W, Wang H, Yang Y, Warsi Z, Asgharzadeh S (2004). Natural killer T cells infiltrate neuroblastomas expressing the chemokine CCL2. J Exp Med.

[20] Lundgren S, Warfvinge CF, Elebro J, Heby M, Nodin B, Krzyzanowska A (2016). The prognostic impact of NK/NKT cell density in periampullary adenocarcinoma differs by morphological type and adjuvant treatment. PLoS ONE.

[21] Tachibana T (2005). Increased intratumor Vα24-positive natural killer T cells: a prognostic factor for primary colorectal carcinomas. Clin Cancer Res.

[22] Gebremeskel S, Lobert L, Tanner K, Walker B, Oliphant T, Clarke LE (2017). Natural killer T-cell immunotherapy in combination with chemotherapy-induced immunogenic cell death targets metastatic breast cancer. Cancer Immunol Res.

[23] Gebremeskel S, Nelson A, Walker B, Oliphant T, Lobert L, Mahoney D (2021). Natural killer T cell immunotherapy combined with oncolytic vesicular stomatitis virus or reovirus treatments differentially increases survival in mouse models of ovarian and breast cancer metastasis. J Immunother Cancer.

[24] Ammayappan A, Peng K-W, Russell SJ (2013). Characteristics of oncolytic vesicular stomatitis virus displaying tumor-targeting ligands. J Virol.

[25] Stojdl DF, Lichty BD, TenOever BR, Paterson JM, Power AT, Knowles S (2003). VSV strains with defects in their ability to shutdown innate immunity are potent systemic anti-cancer agents. Cancer Cell.

[26] Shmulevitz M, Marcato P, Lee PWK (2005). Unshackling the links between reovirus oncolysis, Ras signaling, translational control and cancer. Oncogene.

[27] Lichty BD, Breitbach CJ, Stojdl DF, Bell JC (2014). Going viral with cancer immunotherapy. Nat Rev Cancer.

[28] Lemos de Matos A, Franco LS, McFadden G (2020). Oncolytic viruses and the immune system: the dynamic duo. Mol Ther Methods Clin Dev.

[29] Nelson A, Gebremeskel S, Lichty BD, Johnston B (2022). Natural killer T cell immunotherapy combined with IL-15-expressing oncolytic virotherapy and PD-1 blockade mediates pancreatic tumor regression. J Immunother Cancer.

[30] Ahmed M, McKenzie MO, Puckett S, Hojnacki M, Poliquin L, Lyles DS (2003). Ability of the matrix protein of vesicular stomatitis virus to suppress beta interferon gene expression is genetically correlated with the inhibition of host RNA and protein synthesis. J Virol.

[31] Shen W, Patnaik MM, Ruiz A, Russell SJ, Peng K-W (2016). Immunovirotherapy with vesicular stomatitis virus and PD-L1 blockade enhances therapeutic outcome in murine acute myeloid leukemia. Blood.

[32] VanSeggelen H, Tantalo DGM, Afsahi A, Hammill JA, Bramson JL. Chimeric antigen receptor-engineered T cells as oncolytic virus carriers. Mol Ther - Oncolytics. 2015;2. 10.1038/mto.2015.14. 10.1038/mto.2015.14 PMC4782951 27119109

[33] Lawson ND, Stillman EA, Whitt MA, Rose JK (1995). Recombinant vesicular stomatitis viruses from DNA. Proc Natl Acad Sci U S A.

[34] Le Boeuf F, Gebremeskel S, McMullen N, He H, Greenshields AL, Hoskin DW (2017). Reovirus FAST protein enhances vesicular stomatitis virus oncolytic virotherapy in primary and metastatic tumor models. Mol Ther Oncolytics.

[35] Ciechonska M, Duncan R, Reovirus (2014). FAST proteins: virus-encoded cellular fusogens. Trends Microbiol.

[36] Duncan R (2019). Fusogenic reoviruses and their fusion-associated small transmembrane (FAST) proteins. Annu Rev Virol.

[37] Salsman J, Top D, Boutilier J, Duncan R (2005). Extensive syncytium formation mediated by the reovirus FAST proteins triggers apoptosis-induced membrane instability. J Virol.

[38] Top D, de Antueno R, Salsman J, Corcoran J, Mader J, Hoskin D (2005). Liposome reconstitution of a minimal protein-mediated membrane fusion machine. EMBO J.

[39] Salsman J, Top D, Barry C, Duncan R (2008). A virus-encoded cell–cell fusion machine dependent on surrogate adhesins. PLOS Pathog.

[40] Racine T, Hurst T, Barry C, Shou J, Kibenge F, Duncan R (2009). Aquareovirus effects syncytiogenesis by using a novel member of the FAST protein family translated from a noncanonical translation start site. J Virol.

[41] Le Boeuf F, Diallo J-S, McCart JA, Thorne S, Falls T, Stanford M (2010). Synergistic interaction between oncolytic viruses augments tumor killing. Mol Ther.

[42] Friedrich J, Eder W, Castaneda J, Doss M, Huber E, Ebner R (2007). A reliable tool to determine cell viability in complex 3-D culture: the acid phosphatase assay. J Biomol Screen.

[43] Perry JL, Ramachandran NK, Utama B, Hyser JM (2015). Use of genetically-encoded calcium indicators for live cell calcium imaging and localization in virus-infected cells. Methods.

[44] Stringer C, Wang T, Michaelos M, Pachitariu M (2021). Cellpose: a generalist algorithm for cellular segmentation. Nat Methods.

[45] Bootman MD, Rietdorf K, Collins T, Walker S, Sanderson M (2013). Ca2+-sensitive fluorescent dyes and intracellular Ca2 + imaging. Cold Spring Harb Protoc.

[46] Pulaski BA, Ostrand-Rosenberg S. Mouse 4T1 breast tumor model. Curr Protoc Immunol. 2000;39:20.2.16. 10.1002/0471142735.im2002s39 10.1002/0471142735.im2002s39 18432775

[47] Enot DP, Vacchelli E, Jacquelot N, Zitvogel L, Kroemer G, TumGrowth (2018). An open-access web tool for the statistical analysis of tumor growth curves. Oncoimmunology.

[48] Ahmed M, Puckett S, Lyles DS (2010). Susceptibility of breast cancer cells to an oncolytic matrix (M) protein mutant of vesicular stomatitis virus. Cancer Gene Ther.

[49] Galluzzi L, Buqué A, Kepp O, Zitvogel L, Kroemer G (2017). Immunogenic cell death in cancer and infectious disease. Nat Rev Immunol.

[50] Panaretakis T, Kepp O, Brockmeier U, Tesniere A, Bjorklund A-C, Chapman DC (2009). Mechanisms of pre-apoptotic calreticulin exposure in immunogenic cell death. EMBO J.

[51] Kepp O, Menger L, Vacchelli E, Locher C, Adjemian S, Yamazaki T (2013). Crosstalk between ER stress and immunogenic cell death. Cytokine Growth Factor Rev.

[52] Ciechonska M, Key T, Duncan R (2014). Efficient reovirus- and measles virus-mediated pore expansion during syncytium formation is dependent on annexin A1 and intracellular calcium. J Virol.

[53] Chang-Graham AL, Perry JL, Engevik MA, Engevik KA, Scribano FJ, Gebert JT (2020). Rotavirus induces intercellular calcium waves through ADP signaling. Science.

[54] Obeid M, Tesniere A, Ghiringhelli F, Fimia GM, Apetoh L, Perfettini J-L (2007). Calreticulin exposure dictates the immunogenicity of cancer cell death. Nat Med.

[55] Tufi R, Panaretakis T, Bianchi K, Criollo A, Fazi B, Di Sano F (2008). Reduction of endoplasmic reticulum Ca2 + levels favors plasma membrane surface exposure of calreticulin. Cell Death Differ.

[56] Apetoh L, Ghiringhelli F, Tesniere A, Obeid M, Ortiz C, Criollo A (2007). Toll-like receptor 4–dependent contribution of the immune system to anticancer chemotherapy and radiotherapy. Nat Med.

[57] Sistigu A, Yamazaki T, Vacchelli E, Chaba K, Enot DP, Adam J (2014). Cancer cell–autonomous contribution of type I interferon signaling to the efficacy of chemotherapy. Nat Med.

[58] Patel MR, Dash A, Jacobson BA, Ji Y, Baumann D, Ismail K (2019). JAK/STAT inhibition with ruxolitinib enhances oncolytic virotherapy in non-small cell lung cancer models. Cancer Gene Ther.

[59] Brown C, Stephenson KB, Hanson S, Kucharczyk M, Duncan R, Bell JC (2009). The p14 FAST protein of reptilian reovirus increases vesicular stomatitis virus neuropathogenesis. J Virol.

[60] Burton C, Bartee E (2019). Syncytia formation in oncolytic virotherapy. Mol Ther - Oncolytics.

[61] Bateman AR, Harrington KJ, Kottke T, Ahmed A, Melcher AA, Gough MJ (2002). Viral fusogenic membrane glycoproteins kill solid tumor cells by nonapoptotic mechanisms that promote cross presentation of tumor antigens by dendritic cells. Cancer Res.

[62] Hoffmann D, Bayer W, Wildner O (2007). Therapeutic immune response induced by intratumoral expression of the fusogenic membrane protein of vesicular stomatitis virus and cytokines encoded by adenoviral vectors. Int J Mol Med.

[63] Yang Y, Gaspard G, McMullen N, Duncan R. Polycistronic genome segment evolution and gain and loss of FAST protein function during fusogenic orthoreovirus speciation. Viruses 2020;12:702 10.3390/v12070702 10.3390/v12070702 PMC7412057 32610593

[64] Clancy EK, Duncan R (2011). Helix-destabilizing, β-branched, and polar residues in the baboon reovirus p15 transmembrane domain influence the modularity of FAST proteins. J Virol.

[65] Clancy EK, Duncan R (2009). Reovirus FAST protein transmembrane domains function in a modular, primary sequence-independent manner to mediate cell-cell membrane fusion. J Virol.

[66] Corcoran JA, Syvitski R, Top D, Epand RM, Epand RF, Jakeman D (2004). Myristoylation, a protruding loop, and structural plasticity are essential features of a nonenveloped virus fusion peptide motif. J Biol Chem.

[67] Sandra D, CJ A, CE K, Jayme S, Roy D (2005). Unusual topological arrangement of structural motifs in the baboon reovirus fusion-associated small transmembrane protein. J Virol.

[68] Galivo F, Diaz RM, Wongthida P, Thompson J, Kottke T, Barber G (2010). Single-cycle viral gene expression, rather than progressive replication and oncolysis, is required for VSV therapy of B16 melanoma. Gene Ther.

[69] Prestwich RJ, Ilett EJ, Errington F, Diaz RM, Steele LP, Kottke T (2009). Immune-mediated antitumor activity of reovirus is required for therapy and is independent of direct viral oncolysis and replication. Clin Cancer Res.

[70] Zhang J, Tai L-H, Ilkow CS, Alkayyal AA, Ananth AA, de Souza CT (2014). Maraba MG1 virus enhances natural killer cell function via conventional dendritic cells to reduce postoperative metastatic disease. Mol Ther.

[71] Bonaventura P, Shekarian T, Alcazer V, Valladeau-Guilemond J, Valsesia-Wittmann S, Amigorena S (2019). Cold tumors: a therapeutic challenge for immunotherapy. Front Immunol.

[72] Wang Q, Yuan X, Chen Y, Zheng Q, Xu L, Wu Y. Endoplasmic reticulum stress mediated MDRV p10.8 protein-induced cell cycle arrest and apoptosis through the PERK/eIF2α pathway. Front Microbiol 2018;9:1327. 10.3389/fmicb.2018.01327 10.3389/fmicb.2018.01327 PMC6021497 29977231

[73] Higuchi H, Bronk SF, Bateman A, Harrington K, Vile RG, Gores GJ (2000). Viral fusogenic membrane glycoprotein expression causes syncytia formation with bioenergetic cell death: implications for gene therapy. Cancer Res.

[74] Dumitriu IE, Bianchi ME, Bacci M, Manfredi AA, Rovere-Querini P (2007). The secretion of HMGB1 is required for the migration of maturing dendritic cells. J Leukoc Biol.

[75] Johnston B, Kim CH, Soler D, Emoto M, Butcher EC (2003). Differential chemokine responses and homing patterns of murine TCRαβ NKT cell subsets. J Immunol.

[76] Macho-Fernandez E, Cruz LJ, Ghinnagow R, Fontaine J, Bialecki E, Frisch B (2014). Targeted delivery of α-galactosylceramide to CD8α^+^ dendritic cells optimizes type I NKT cell–based antitumor responses. J Immunol.

[77] Dent R, Trudeau M, Pritchard KI, Hanna WM, Kahn HK, Sawka CA (2007). Triple-negative breast cancer: clinical features and patterns of recurrence. Clin Cancer Res.

[78] Russell SJ, Peng K-W, Bell JC (2012). Oncolytic virotherapy. Nat Biotechnol.

[79] Nair S, Dhodapkar MV (2017). Natural killer T cells in cancer immunotherapy. Front Immunol.

