# Supplementary figures and images for: Fusogenic vesicular stomatitis virus combined with natural killer T cell immunotherapy controls metastatic breast cancer
Source: Breast Cancer Res. 2024 May 15;26:78. doi: 10.1186/s13058-024-01818-5 (PMC11094881; doi:10.1186/s13058-024-01818-5)

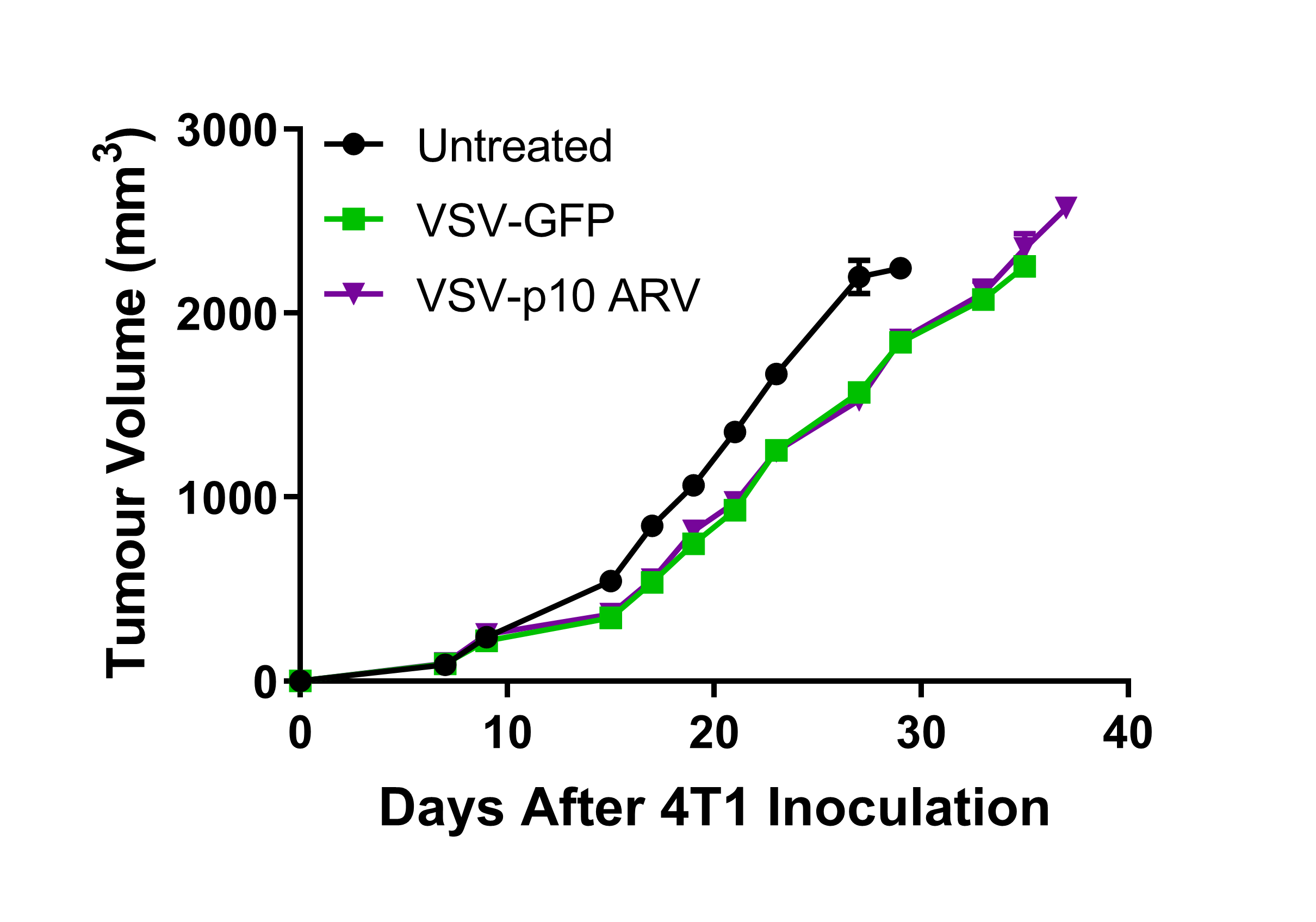

Supplement: Supplementary file 1 — Supplementary Material 1: Sup. Fig 1: p10ARV does not increase activity of VSV and overall survival in a primary 4T1 model. 4T1 tumor volume was assessed in untreated tumor-bearing mice and mice treated with108PFU VSV-GFP or VSV-p10ARV administered following the same timeline as Fig 2A (n= 10-14 per group). Untreated and VSV-GFP data are shown from figure 2B. [file 13058_2024_1818_MOESM1_ESM.tif]

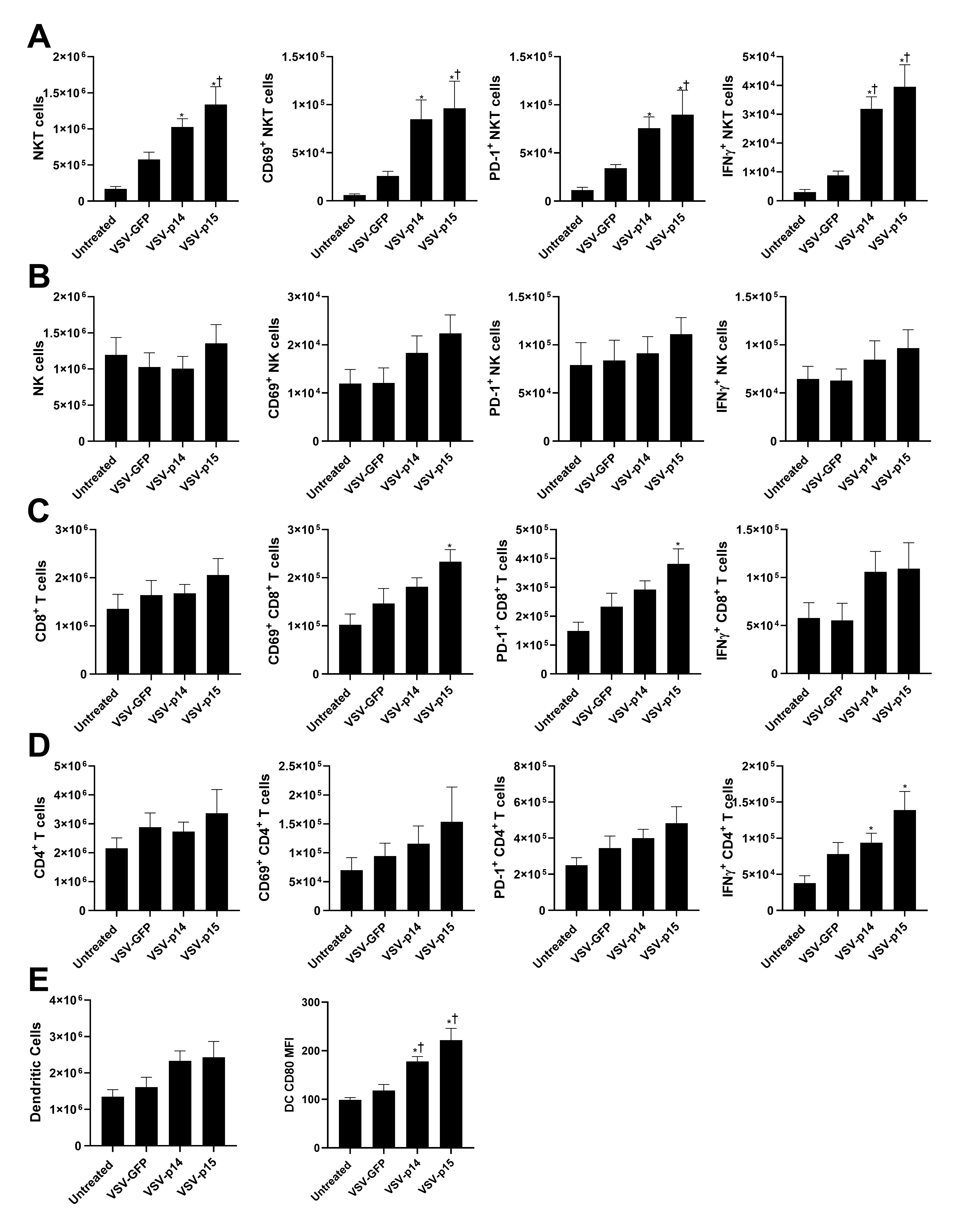

Supplement: Supplementary file 2 — Supplementary Material 2: Sup. Figure 2: VSV-FAST constructs increase immune activation in the spleen in a primary 4T1 tumor model. Spleens from untreated tumor-bearing mice and treated mice were isolated and dispersed into single cell suspensions. Flow cytometry was used to assess immune cellexpansion and activation in the spleen seven days after the end of treatment (n= 8-9 per group). The number of A) NKT cells (CD1d tetramer+ TCRβ+), B) NK cells (NK1.1+ TCRβ-) C) CD8+ T cells (TCRβ+ CD8α+), D) CD4+ T cells (TCRβ+ CD4+) and the expression of CD69, PD-1, and intracellular IFNγ by these subsets was assessed. E) The number of dendritic cells (MHC II+CD11c+) and CD80 expression was also examined. *p<0.05compared to untreated, †P<0.05 compared to VSV-GFP. [file 13058_2024_1818_MOESM2_ESM.tif]
